# Extracellular vesicles: innovative cell-free solutions for wound repair

**DOI:** 10.3389/fbioe.2025.1571461

**Published:** 2025-04-03

**Authors:** Hanne Eerdekens, Elke Pirlet, Sarah Willems, Annelies Bronckaers, Paula M. Pincela Lins

**Affiliations:** ^1^ Hasselt University, Faculty of Medicine and Life Sciences, Biomedical Research Institute (BIOMED), Diepenbeek, Belgium; ^2^ Flemish Institute for Technological Research (VITO), Environmental Intelligence Unit, Mol, Belgium

**Keywords:** wound healing, exosomes, mesenchymal stromal cells, skin biology, wound biology extracellular vesicles

## Abstract

Chronic non-healing wounds are often associated with conditions such as diabetes and peripheral vascular disease, pose significant medical and socioeconomic challenges. Cell-based therapies have shown promise in promoting wound healing but have major drawbacks such as immunogenicity and tumor formation. As a result, recent research has shifted to the potential of extracellular vesicles (EVs) derived from these cells. EVs are nanosized lipid bilayer vesicles, naturally produced by all cell types, which facilitate intercellular communication and carry bioactive molecules, offering advantages such as low immunogenicity, negligible toxicity and the potential to be re-engineered. Recent evidence recognizes that during wound healing EVs are released from a wide range of cells including immune cells, skin cells, epithelial cells and platelets and they actively participate in wound repair. This review comprehensively summarizes the latest research on the function of EVs from endogenous cell types during the different phases of wound healing, thereby presenting interesting therapeutic targets. Additionally, it gives a critical overview of the current status of mesenchymal stem cell-derived EVs in wound treatment highlighting their tremendous therapeutic potential as a non-cellular of-the-shelf alternative in wound care.

## 1 Introduction

Various pathologies lead to inadequate wound healing, including peripheral vascular disease, diabetes mellitus, cancer, and ischemia (Veith et al., 2019). Despite recent medical advances, non-healing, chronic wounds have become a major medical and social burden worldwide. For example, patients with diabetes suffer from multiple complications during their lifetime, with diabetic foot ulcers (DFUs) being one of the most serious ones. Astonishingly, DFUs are responsible for 25%–50% of the total cost of diabetes treatment and are the most common cause of limb amputations (Veith et al., 2019). These wounds are one of the largest reasons for hospitalization and mortality among diabetic patients. Across Europe, 1.5–2 million people are living with a chronic wound, while in the USA 6.5 million people are affected (Lindholm and Searle, 2016). Consequently, new treatments are urgently needed.

Replacing the damaged tissue with stem cells or skin-derived cell types such as endothelial cells, has been a proven successful approach in fostering wound healing in a preclinical setting (Phinney and Pittenger, 2017). Nevertheless, the clinical applications of cells are limited. In general, cell-based therapy has severe disadvantages such as potential immunogenicity, tumor formation and uncertain dosing. Another burden is reaching sufficient numbers of cells due to senescence (Medina et al., 2013; Jang et al., 2013; Musiał-Wysocka et al., 2019). Hence, recent research interest has shifted towards the use of extracellular vesicles (EVs) as an alternative.

EVs are small, lipid bilayer vesicles that are naturally produced by all cell types and aid in intercellular communication by carrying a wide variety of bioactive molecules such as lipids, metabolites, proteins and nucleic acids (Kalluri and LeBleu, 2020; Welsh et al., 2024). They pose several advantages, including low immunological rejection, negligible toxicity, and the ability to be re-engineered or combined with drugs and other biomaterials. Indeed, the number of articles exploring the use of EVs for wound healing has rapidly soared (Phinney and Pittenger, 2017; Narauskaitė et al., 2021), indicating the need for a clear overview. In the present review, we discuss the physiology of the wounded skin, the biogenesis of EVs, their mode of action and their endogenous role in various stages of wound healing. In addition, we provide a detailed overview of the current preclinical and clinical status of the application of mesenchymal stem cell (MSC)-derived EVs for the treatment of wounds.

## 2 Physiology of healthy and wounded skin

The skin is the largest organ in the human body, serving as a complex and versatile protective barrier between the internal organs and the external environment. Its intricate structure plays a pivotal role in maintaining homeostasis and safeguarding the body against various external threats, such as ultraviolet radiation, chemicals, microorganisms, mechanical forces, and extreme weather conditions (Rodrigues et al., 2019). Despite its resilience, the skin is susceptible to damage, and the loss of skin integrity can result in severe injuries. Consequently, efficient wound repair is vital for restoring the skin’s protective function and ensuring the overall health of the body.

Successful wound healing relies on a coordinated response between the three skin layers: the epidermis, dermis, and hypodermis. The outermost layer, the epidermis, serves as a waterproof barrier and regenerates continuously. Beneath it lies the dermis, a thicker layer containing blood vessels, nerve endings, hair follicles, and sweat glands, providing structural support, elasticity, and nourishment (Rodrigues et al., 2019; Evans et al., 2013). The deepest layer, the hypodermis, consists mainly of adipose tissue that serves as insulation and energy storage (Gilaberte LP-T et al., 2016). When damage occurs to these skin layers, a swift process of wound healing is initiated to reinstate the skin’s integrity and functionality.

The wound healing process is categorized into four overlapping stages: hemostasis, inflammation, proliferation, and remodeling. The initial response to skin injury is hemostasis, which prevents excessive bleeding from damaged blood vessels. This process promptly triggers vasoconstriction to limit blood loss, followed by platelet adhesion to exposed collagen in the subendothelial matrix and platelet clot formation. Subsequently, the coagulation cascade is activated, converting fibrinogen to fibrin, which intertwines with the platelet clot to form a thrombus. This latter stops the bleeding, releases growth factors and serves as a scaffold for infiltrating immune cells (Rodrigues et al., 2019; Wilkinson and Hardman, 2020).

The inflammatory phase is initiated by the massive influx of immune cells. Neutrophils are quickly recruited to the injury site by signals from necrotic cells, collectively termed damage-associated molecular patterns (DAMPs) or components from pathogens collectively termed pathogens-associated molecular patterns (PAMPs). These phagocytes clear debris and eliminate pathogens through various mechanisms, such as toxic granules, oxidative burst, and neutrophil extracellular traps (Wilkinson and Hardman, 2020; Ellis et al., 2018). Subsequently, blood monocytes enter the wound and undergo a phenotypic change to pro-inflammatory ‘M1’ macrophages upon exposure to DAMPs and PAMPs. These macrophages also have a phagocytic function and amplify inflammation by releasing pro-inflammatory cytokines such as Tumor necrosis factor-α, interleukin (IL)-6, IL-1β and recruiting even more immune cells to the wound. As the healing process progresses, a shift towards anti-inflammatory ‘M2’ macrophages occurs, leading to the release of anti-inflammatory cytokines and growth factors, which provides a supportive environment for the proliferation phase and tissue repair (Ellis et al., 2018; Canedo-Dorantes and Canedo-Ayala, 2019).

In the proliferation phase, the initial fibrin matrix formed during hemostasis is replaced by granulation tissue, which facilitates effective wound closure and comprises fibroblasts, endothelial cells, and immune cells. Fibroblasts produce proteinases to degrade the fibrin matrix and participate in the synthesis of extracellular matrix (ECM) components (such as collagen, proteoglycans, and hyaluronic acid) to provide essential structural tissue support (Landen et al., 2016). Concurrently, the release of VEGF in the wound initiates angiogenesis, which is the formation of new blood vessels. These vessels permeate the granulation tissue, ensuring an adequate supply of blood, oxygen and nutrients to the healing tissue. Additionally, wound-resident immune cells contribute to a robust defense against potential external microbial threats. Moreover, the granulation tissue also serves as a stable scaffold for keratinocyte migration during reepithelization of the wound area (Landen et al., 2016; Enoch and Leaper, 2008).

The final stage of wound healing is the remodeling phase and is characterized by the refinement and strengthening of the healing tissue. In this stage, fibroblasts actively remodel the ECM, boosting the overall tensile strength of the tissue. Simultaneously, blood vessels undergo pruning to optimize blood flow to the healed tissue, while the resolution of inflammation contributes to improved tissue stability. Additionally, collagen synthesis naturally leads to scar formation and myofibroblasts (i.e., contractile fibroblasts) aid in reducing wound and scar size through tissue contraction. These processes ultimately contribute to the maturation of a functional scar (Rodrigues et al., 2019; Wilkinson and Hardman, 2020; Enoch and Leaper, 2008).

**FIGURE 1:**
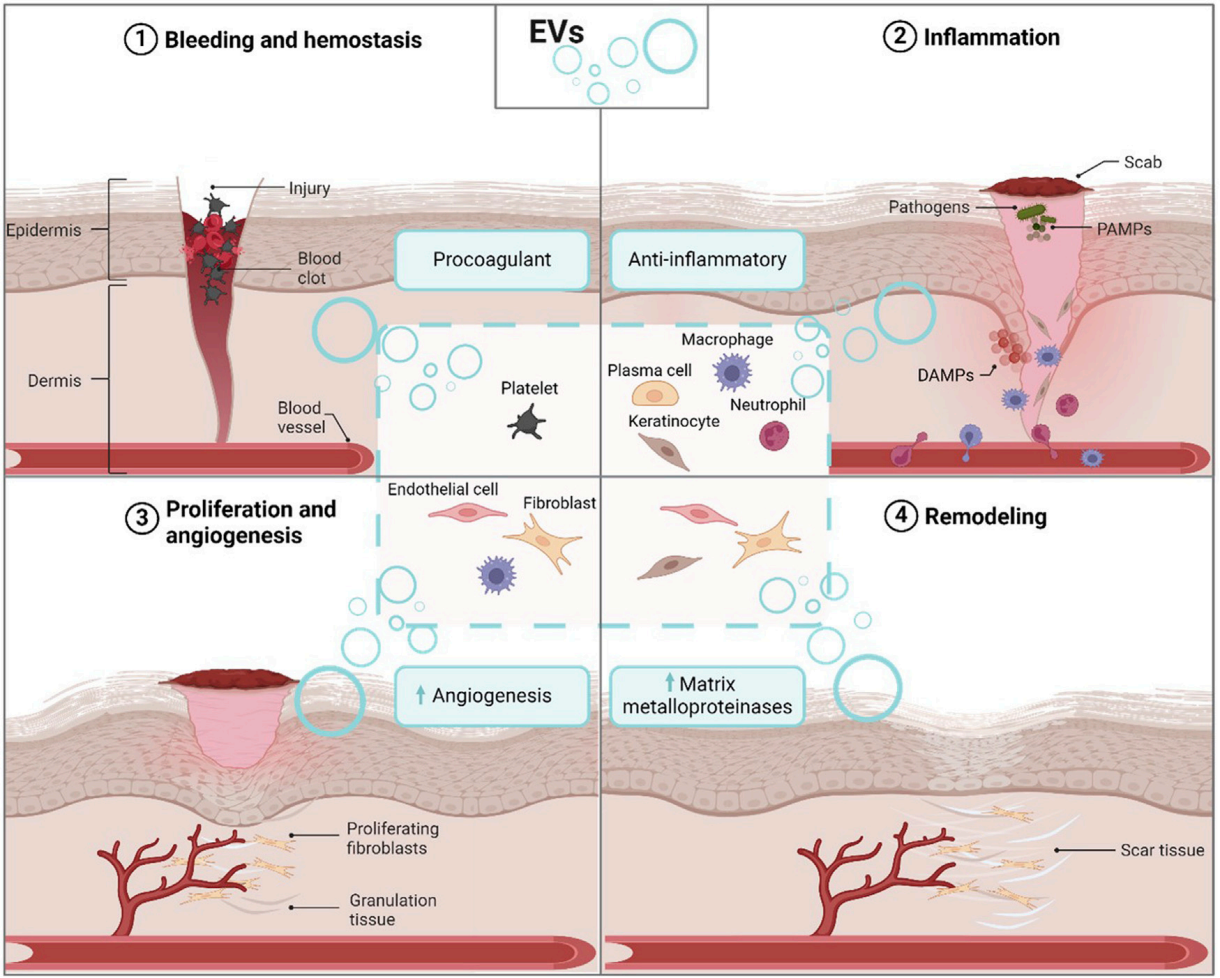
Effect of endogenous cell-derived EVs on wound repair. EVs secreted by various cell types such as platelets, plasma cells, macrophages, keratinocytes, neutrophils, endothelial cells, and fibroblasts, play a diverse role in the four phases of wound healing: hemostasis, inflammation, proliferation, and remodeling. EVs derived from different cellular sources can either stimulate coagulation, anti-inflammation, angiogenesis, or the production of matrix metalloproteinases. Abbreviation: EVs: extracellular vesicles. DAMPs, damage-associated molecular patterns; PAMPs, pathogens-associated molecular patterns. Figure created with biorender.com.

Each stage of the wound healing process is crucial, and their coordinated interactions ensure effective wound closure and functional scar formation. The overview of the wound healing steps is shown in Figure 1. Harmonious collaboration among these four stages is imperative, as any disruption or imbalance to one or more stages can impede the healing process, resulting in complications (e.g., chronic wounds and fibrosis) and delayed recovery. Factors such as infection, poor blood flow, nutritional deficiency, chronic diseases (e.g., diabetes or cardiovascular diseases), medication types, immunocompromised conditions, and age-related factors can significantly affect wound repair and result in chronic wounds or even delayed healing of acute wounds (Rodrigues et al., 2019; Guo and DiPietro, 2010; Monika et al., 2021). Understanding the interplay of these stages and the involvement of various factors facilitates the development of therapeutic strategies that support a more favorable healing environment and ultimately enhance effective wound closure. Furthermore, EVs are emerging as key players in wound healing, delivering vital proteins and genetic materials to stimulate tissue regeneration while modulating inflammatory and healing pathways. This highlights the therapeutic promise of EVs, presenting new opportunities for advancing wound care and treating related skin pathologies such as chronic wounds, fibrosis, and ulcers.

## 3 The biogenesis, composition, and function of extracellular vesicles

### 3.1 The different subpopulations based on the biogenesis of extracellular vesicles

As stated in the introduction, EVs are small, lipid bilayer vesicles that are secreted by cells during health and disease, *in vivo* and *in vitro*. Exosomes, microvesicles and other vesicles are recognized as EV subtypes and this nomenclature is based on their size and biological origin (Akers et al., 2013; Cable et al., 2023).

Exosomes originate from endosomes (Keshtkar et al., 2018; Jeppesen et al., 2023). Their biogenesis initiates through the endocytosis-exocytosis pathway, where small quantities of intracellular fluids in specific membrane regions are engulfed to form early endosomes. These early endosomes progress into late endosomes, where internal budding gives rise to the formation of intraluminal vesicles. Subsequently, multivesicular bodies (MVBs) are generated, derived from the intraluminal vesicles. MVBs fuse with the cell membrane, leading to their release into the extracellular environment, where they are identified as exosomes (Keshtkar et al., 2018; Jeppesen et al., 2023). These exosomes represent a subtype of EVs characterized by a size spectrum ranging from approximately 40–160 nm with an average diameter of 100 nm (Kalluri, 2016). These dimensions emphasize their potential in various biological processes, including intercellular communication (Kalluri, 2016; Sung et al., 2020).

In contrast, microvesicles, arise by direct outward budding from the plasma membrane (Stahl and Raposo, 2019). This process is based on the reorganization of lipids, and the use of contractile machinery of the plasma membrane to allow vesicles to be released. Microvesicles exhibit a broad size spectrum, ranging from 100 nm to 1 µm in diameter (Zaborowski et al., 2015). The overlapping in EV size of different biogenesis routes underlines the heterogeneity within this EV category, underscoring their diverse roles and functions (Tricarico et al., 2017).

Apoptotic vesicles are vesicles formed during cell-programmed death. When a cell undergoes apoptosis, the cell membrane shrinks, forming blebs and consequently protrusions are released, known as apoptotic bodies, from a size range of 1–5 µm (Dixson et al., 2023). Apoptotic bodies were the first class characterized of apoptotic vesicles, and further investigation showed that these cells also produce smaller-sized vesicles (Tucher et al., 2018; Zou et al., 2023). These apoptotic vesicles endow the ‘eat-me’ signal from apoptotic cells and are in a better size to facilitate the engulfment of phagocytes for immune activation (Poon et al., 2014). They are also known for their capability to carry microbial (Schaible et al., 2003) and tumor antigens (Muhsin-Sharafaldine et al., 2016).

Due to the size overlap and difficulty in differentiating the subpopulations in commonly used isolation methods, the International Society of Extracellular Vesicles (ISEV) latest guidelines are to describe EVs by size as small (<200 nm) and large (>200 nm) (Thery et al., 2018). In this review, we will follow these guidelines unless stated otherwise.

An important concept when it comes to EV research are the ‘Minimal information studies of extracellular vesicles (MISEV)’ guidelines, promoting standardized reporting in separation, characterization, and functional studies, underscoring the importance of consistent reporting in all aspects of EV research. For EVs isolated from cell culture medium (CCM), reporting includes cell culture conditions such as seeding density and vessel type, media compositions, stimulation (if any), CCM collection timepoint, and storage procedures (Welsh et al., 2024). Most importantly, in the recent MISEV guidelines, it is recommended that researchers are constantly updated by task forces related to the source of the conditioned media (Shekari et al., 2023).

### 3.2 Cargo of extracellular vesicles

EVs play a vital role in physiological processes by functioning as carriers for diverse cargo subtypes, including membrane- and cytosolic proteins, lipids, and various types of nucleic acids (Raposo and Stoorvogel, 2013). EVs contain key proteins such as tetraspanins, cytoplasmic proteins, membrane-binding proteins, and annexins (Chen et al., 2021; Raiborg and Stenmark, 2009). The functions of EV proteins are notably diverse. For instance, tetraspanins such as CD9 play a pivotal role in sorting and membrane trafficking, while annexins are involved in membrane exchange and fusion (Chen et al., 2021; Keklikoglou et al., 2019).

EVs do not only contain proteins but also nucleic acids, including types of mRNAs, DNAs, and non-coding RNAs like microRNAs (miRNA, miR) (Chen et al., 2021; Mir and Goettsch, 2020). These RNAs play crucial roles through diverse mechanisms. For instance, EV-delivered mRNAs can be translated within the recipient cell, leading to protein synthesis. Moreover, EV miRNAs are shielded from degradation within the vesicle, facilitating their transportation to target cells where they can function by silencing RNA (Chen et al., 2021; Turchinovich et al., 2011). Interestingly, miR-23a-3p released from senescent fibroblasts accelerates the scratch closure of epidermal keratinocytes (Terlecki-Zaniewicz et al., 2019).

In addition to proteins and nucleic acids, lipids are also identified as cargo of EVs. For instance, lipids such as sphingomyelin, ceramide, phosphatidylserine (PS), and glycosphingolipids, are fundamental components of EVs (Su et al., 2021). The roles of EVs lipid cargo, encompassing both membrane-bound and cytoplasmic lipids, extend to the preservation of membrane integrity and facilitation of intercellular communication (Chiva-Blanch and Badimon, 2019; Vanherle et al., 2020). For example, Vanherle et al. highlighted the regenerative properties of cholesterol found in EVs secreted by repair-associated macrophages in the context of multiple sclerosis (Vanherle et al., 2023).

Recognizing the distinct cargo compositions of microvesicles and exosomes, reflective of both the cell of origin and its pathophysiological state, it becomes apparent that these vesicle subtypes have unique cargo profiles (Dozio and Sanchez, 2017; de Jong et al., 2012). For instance, Dozio et al. investigated EVs subtypes cargo from cells stimulated by tumor necrosis factor-α (TNF-α), an inflammatory inducer. Through their proteomics analysis, it was shown that exosomes were enriched with adhesion and ribosomal proteins, while microvesicles were enriched with mitochondrial and cytoskeletal proteins (Dozio and Sanchez, 2017).

### 3.3 The importance of extracellular vesicles in physiological and pathological conditions

The involvement of EVs in intercellular communication is well-established in both physiological and pathological conditions, playing a crucial role in directing diverse processes. In the context of diseases such as cancer, this aspect of EV function is extensively documented. For example, in lung cancer, the presence of EVs may induce myotube atrophy and adipocyte lipolysis through their engagement in the STAT3 pathway (Hu et al., 2019). Moreover, within the ocular melanoma tumor microenvironment, EVs play vital roles in various oncogenic processes, including metastasis, therapeutic resistance, and immune escape (Piquet et al., 2022). Elevated levels of polymeric immunoglobulin receptor in EVs derived from hepatocellular carcinoma patients are implicated in promoting cancer stemness, tumorigenesis, and metastasis (Tey et al., 2022). Beyond cancer, EVs contribute to other pathologies such as peritoneal fibrosis, chronic obstructive pulmonary disease, and ischemic stroke (Neri et al., 2022; Huang et al., 2023).

In normal physiological conditions, EVs possess crucial functions across various body systems. For instance, skeletal muscle-derived EVs aid in myogenesis, muscle homeostasis, and development, providing essential biochemical cues for skeletal muscle generation (Choi et al., 2016; Yates et al., 2022). In the cardiovascular system, EVs are found to be able to modulate blood pressure by enriching angiotensin receptor type 1 and altering nitric oxide levels, although further investigation is needed to elucidate the precise mechanisms (Yates et al., 2022; Pironti et al., 2015; Good et al., 2020). Platelet-derived EVs contribute significantly to both pro- and anticoagulant activity in the blood, with their surface chemistry exhibiting substantially higher procoagulant activity compared to activated platelets (Tans et al., 1991). Various conditions such as cardiovascular stress, hypoxia, inflammation, and high-fat diet consumption increase platelet EV levels in circulation (Yates et al., 2022). Furthermore, EV research extends to the central nervous system CNS, necessitating traversal of the blood-brain barrier (BBB) (Yates et al., 2022). Early *in vivo* studies demonstrate the bidirectional migration of EVs between the periphery and the brain, facilitating cellular communication across the intact BBB (Yates et al., 2022; Obermeier et al., 2013).

Due to these insights into their capacities for intercellular communication, EVs have been the subject of thorough investigation as a therapeutic mean in many disease states. For example, Rong et al. elucidated the use of neural stem cell-derived small EVs to attenuate apoptosis and neuroinflammation after spinal cord injury. The administration of these specialized EVs resulted in a reduction in the extent of spinal cord injury and an improvement in functional recovery (Rong et al., 2019). Additionally, Adamczyk et al. reported the beneficial effects of EVs isolated from plasma in counteracting the pro-inflammatory state of macrophages during inflammation. This further underscores the crucial role of EVs in regulating diverse processes through intercellular communication (Adamczyk et al., 2023).

## 4 The role of endogenous extracellular vesicles in wound healing

EVs are recognized as key players in the complex process of wound healing. As depicted in Figure 1, they are released from a wide range of cells including immune cells, skin cells, epithelial cells and platelets. Research exploring the effects of EVs on wound healing has significantly grown, uncovering their impact on modulating inflammation, enhancing hemostasis, promoting cell proliferation, regulating remodeling, and aiding in tissue regeneration. This has shed light on their therapeutic potential in the critical physiological process of wound healing, and a summary is shown in Table 1.

**TABLE 1:** The role of native extracellular vesicles in different wound healing phases.

Phase	EVs source	Model/*in vitro*	Effect/*in vivo*	Signaling pathway	Reference
**Hemostasis**	Complement proteins C5b-9 activated platelets	**Platelets:** ↑Formation of thrombin	**NE**	Coagulation factor Va	Sims TW et al. (1989)
	Thrombin activated platelets, collagen activated platelets, and calcium ionophore A23187 activated platelets	**Platelets:** ↑Recruitment of platelets, ↑formation of fibrin	**NE**	Activated form of plasma membrane glycoproteins GP Ib and integrin αIIbβ3	
	Thrombin activated platelets	**Platelets:** ↑Formation of fibrin	**C57BL/6 mice tail-bleedingmodel** ↓Bleeding time and blood loss	Activated form of integrin αIIbβ3	Lee et al. (2020)
	Thrombin activated platelets	**Platelets:** ↑Expression of integrin αIIbβ3	**NE**	Activated form of integrin αIIbβ3	Heinzmann et al. (2020)
	Thrombin activated platelets	**Platelets:** ↑Expression of integrin αIIbβ3, plasma membrane glycoproteins GP Ib, and P-selectin	**NE**	Activated form of integrin αIIbβ3, plasma membrane glycoproteins GP Ib, and P-selectin	Heijnen et al. (1999)
	ADP activated platelets	**Platelets:** ↑Expression of, P-selectin and PS **Plasma:** ↑Formation of fibrin	**NE**	Activated form of P-selectin and PS	Gasecka et al. (2019)
**Proliferation and angiogenesis**	Endothelial progenitor cells	**HMEC-1:** ↑Proliferation, migration, and tube formation, ↑Angiogenesis-related gene expression of FGF-2, IL-6, IL-8, c-Myc, Id1, Cox-2, VEGFA, E-selectin, and angiopoietin	**Sprague-Dawley rats diabetic excisional wound model:** ↑Re-epithelialization, collagen maturity, new blood vessels formation, ↓Scar formation	Erk1/2 signaling pathway	Zhang et al. (2016a)
	Endothelial progenitor cells	**HUVECs:** ↑Proliferation, migration, and tube formation	**Sprague-Dawley rats acute lung injury model:** Alleviated LPS-induced lung injury	RAF/ERK signaling pathway	Wu et al. (2018)
	Bone marrow endothelial progenitor cells	**NE**	**Healthy and diabetic** **C57BL/6 mice excisional wound model:** ↑ Skin wound healing ↑VEGF, PECAM-1, and Ki67	AGE-RAGE signaling pathway	Xu et al. (2020)
	Bone marrow endothelial progenitor cells	**HUVECs:** ↑Proliferation, migration, and tube formation, ↑angiogenesis-related gene expression of HIF-1α, VEGF, FGF-2, TGF-β1, and ANG	**Sprague-Dawley rats unilateral tibial DO model:** Bone regeneration was markedly accelerated, ↑vessel density, ↑angiogenesis	RAF/ERK signaling pathway	Jia et al. (2019)
	Macrophages	**HUVECs:** ↑Migration, proliferation, and matrigel tube formation	**Sprague-Dawley rats diabetic model:** ↓Diabetic wound size and neutrophils/macrophages infiltration ↑collagen deposition, re-epithelialization,↑neovascularization	AKT/VEGF pathway	Li et al. (2019b)
	M2 macrophages	**HUVECs:** ↑Migration, proliferation, and matrigel tube formation	**BALB/c mice excisional wound model:** ↑Wound closure, ↑collagen deposition, ↑IL-10 expression, ↓IL-1β and TNF-α, ↑angiogenesis-related gene expression of VEGF and TGF- β	Inhibition of PTEN AKT/mTOR pathway	Lyu et al. (2022)
**Inflammation**	Bone marrow derived-IL-4 activated—M2 macrophages	**Bone marrow derived-IFN-γ activated—M1 macrophages:** ↓iNOS and ↑arginase-1 markers **NIH-3T3 fibroblasts:** ↑Fibroblast migration and proliferation **SVEC4-10 endothelial cells:** ↑Tube formation	**BALB/c mice excisional wound model:** ↓iNOS and ↑arginase-1, ↑wound closure, ↑angiogenesis, ↑re-epithelialization, ↑collagen deposition.	CCL24, CCL22, and MFG-E8 cytokines	Kim et al. (2019)
	Mouse wound edge keratinocytes	**Bone marrow derived macrophages:** Glycan ions with high mannose, Macrophages polarization from M1 to M2, ↓NOS2, ↓CD74, ↓TNF-α, ↑CCL3	**C57BL/6 mice excisional wound splinting model:** ↓Accumulation of macrophages, ↓iNOS M1 and ↑arginase M2 markers, ↑Skin barrier-function	Not determined	Zhou et al. (2020a)
	Human plasma	**Human monocyte-derived macrophages:** ↑CD163, CD206 and merTK M2 markers; ↓pro-inflammatory IL-6 and TNF-α; ↑anti-inflammatory IL-10 **HUVECs:** ↑Tube formation; ↑VEGFa, CD300e, RGS2, and CD93 expression	**NE**	PGE2/CREB pathway	Adamczyk et al. (2023)
	Opsonized zymosan A activated neutrophils	**Neutrophils:** ↑ROS and IL-8 **HUVECs:** ↑E-selectin, and VCAM1	**NE**	Not determined	Kolonics et al. (2021)
	Resting state neutrophils	**Neutrophils:** ↓ROS and IL-8 **HUVECs:** ↑Coagulation	**NE**	Not determined	
**Skin remodeling**	Mouse fibroblast cell line	**Mouse fibroblast cell line:** ↑Fibroblast migration and proliferation, ↑*MMP-1, MMP-3*, COL3A1, and collagen I gene expression **Endothelial cell line:** ↑Migration and tube formation	**C57BL/6 mice excisional wound splinting model:** ↑Wound healing, ↑collagen formation, ↑collagen maturation, ↑blood vessels, ↑angiogenesis	Not determined	Oh et al. (2021)
	HMEC-1	**Human** **annulus fibrosus:** ↑MMP-1, MMP-3, and MMP-13 gene and protein expression **HMEC-1:** ↑Migration	**NE**		Pohl et al. (2016)
	HMEC-1	**Human dermal fibroblasts:** ↑LOX activity, ↑fibroblast activation, ↑Collagen gel contraction	**NE**	LOXL-2	de Jong et al. (2016)
	Human epidermal keratinocyte adult	**Human foreskin fibroblasts:** ↑Migration, fibroblast-mediated endothelial tube formation, ↑MMP-1, MMP-3, TIMP3, and TIMP4 expression, ↑α-SMA and N-cadherin	**Sprague-Dawley rats diabetic excisional wound model:** ↑Wound healing	miR-21 and ERK1/2 pathway	Li et al. (2019a)

AGE-RAGE, Advanced Glycation End-products and Receptor for Advanced Glycation End-products; AKT, Protein Kinase B; ANG, angiopoietin; C5b-9, Complement component 5b-9; CCL22, Chemokine (C-C motif) ligand 22; CCL24, Chemokine (C-C motif) ligand 24; COL3A1, Collagen Type III, Alpha 1 Chain; CREB, cAMP, Response Element-Binding protein; ERK1/2, Extracellular Signal-Regulated Kinases 1/2; FGF-2, Fibroblast Growth Factor-2; GP, ib, Glycoprotein Ib; HIF-1α, Hypoxia-Inducible Factor 1-alpha; HMEC-1, Human Microvascular Endothelial Cell line-1; HUVECs, Human umbilical vein endothelial cells; IL-6, Interleukin-6; IL-8, Interleukin-8; iNOS, inducible nitric oxide synthase; LOX, lysyl oxidase; LOXL-2, Lysyl Oxidase-Like 2; M2 macrophages, pro-resolving/healing macrophage type; MFG-E8, Milk Fat Globule-EGF, Factor 8 Protein; miR-21, MicroRNA-21; MMP-1, Matrix Metallopeptidase 1; MMP-3, Matrix Metallopeptidase 3; mTOR, mammalian target of rapamycin; NE, not evaluated; PECAM-1, Platelet Endothelial Cell Adhesion Molecule-1; PGE2, Prostaglandin E2; PS, phosphatidylserine; PTEN, phosphatase and tensin homolog; RAF/ERK, RAF Kinase/Extracellular Signal-Regulated Kinases; ROS, reactive oxygen species; TGF-β1, Transforming Growth Factor Beta 1; TIMP3, Tissue Inhibitor of Metalloproteinases 3; TIMP4, Tissue Inhibitor of Metalloproteinases 4; VCAM-1, Vascular Cell Adhesion Molecule 1; VEGF, vascular endothelial growth factor; VEGFA, Vascular Endothelial Growth Factor A; α-SMA, Alpha-Smooth Muscle Actin; αIIbβ3, Integrin alpha-IIb, beta-3; ↑, increased; ↓, decreased.

### 4.1 Promoting hemostasis

Hemostasis is a natural process of the body that involves a sequence of tightly regulated events aimed at maintaining blood vessel integrity and preventing excessive blood loss after injury (Versteeg et al., 2013). Platelet-derived EVs (P-EVs) are the most abundant EVs in blood circulation and contribute to hemostasis. (Versteeg et al., 2013; Kerris et al., 2020). Upon skin injury, platelets become activated prompted by the release of various danger-signaling molecules including a combination of collagen and thrombin, complement proteins C5b-9, calcium ionophore, as well as specific individual triggers such as thrombin, ADP, or epinephrine. Next, platelets adhere to the site of injury on the damaged blood vessel wall, where they stick to exposed collagen and other components of the injured blood vessel to temporarily stop the bleeding (Kerris et al., 2020; LaPelusa and Dave, 2024). Upon activation by a variety of agonists, platelets readily generate EVs, which were initially identified as procoagulant particles (Chaudhary et al., 2023). Sims et al. showed that activation of human platelets by complement proteins C5b-9 is accompanied by the release of P-EVs, which are highly enriched in binding sites for coagulation factor Va and exhibit prothrombinase activity (Sims TW et al., 1989). In addition, this research indicated that thrombin, collagen, and the calcium ionophore A23187 were each found to induce the formation of platelet microvesicles that incorporated plasma membrane glycoproteins (GP) Ib and GP IIb/IIIa, also known as integrin αIIbβ3, which has an essential role in cross-linking of adjacent platelets, leading to the formation of stable blood clots (Lee et al., 2020; Heinzmann et al., 2020; Heijnen et al., 1999). Another mechanism by which P-EVs contribute to clot formation is through the fact that they contain activated P-selectin as cargo (Heijnen et al., 1999). P-selectin is known to play a pivotal role in the initial interactions between platelets and the damaged blood vessel walls. Additionally, exposure of the pro-inflammatory P-selectin and procoagulant PS on P-EVs serves as a binding site for various coagulation factors, such as factor X and prothrombin, facilitating their assembly into complexes that accelerate the conversion of prothrombin to thrombin. Thrombin facilitates wound healing in all the stages by promoting blood clot formation, activating immune cells, and stimulating tissue repair processes such as angiogenesis (Gasecka et al., 2019).

### 4.2 Enhancing proliferation and angiogenesis

EVs play a pivotal role during the proliferation phase of wound healing. When there is an injury, the site often experiences low oxygen levels. This lack of oxygen creates a state called hypoxia which triggers the activation of local vascular endothelial cells, thereby inducing blood vessel formation or angiogenesis. Numerous studies have underscored the pivotal role of EVs sourced from diverse cell types, including endothelial progenitor cells (EPCs), macrophages, and fibroblasts, in the angiogenesis process.

Zhang et. al. demonstrated the potential of EPC-EVs in promoting wound healing. *In vitro* experiments showed increased proliferation, migration, and tube formation of human microvascular endothelial cells (HMEC-1) when exposed to EPC-EVs. *In vivo* studies using diabetic skin wounds in adult male rats revealed enhanced re-epithelialization, reduced scar formation, increased collagen maturity, and formation of new blood vessels after local transplantation of EPC-EVs via subcutaneous injection. Furthermore, EPC-EV treatment led to significant alterations in the expression levels of genes involved in the Erk1/2 signaling pathway as the fibroblast growth factor 2 (FGF-2), IL-6, and IL-8. By inhibiting this pathway in HMEC-1, the EPC-EVs stimulation showed lower angiogenic effects. Subsequent angiogenesis assays confirmed the pivotal role of Erk1/2 signaling in mediating EPC-EVs-induced pro-angiogenic effects on HMEC-1 (Zhang J. et al., 2016). Another study corroborated these findings, demonstrating enhanced endothelial cell proliferation, migration, and tube formation after treatment with EPC-EVs, using human umbilical vein endothelial cells (HUVECs). Additionally, their therapeutic potential was validated in a model of acute pulmonary injury, where intravenously administered EPC-EVs increased angiogenesis, ameliorated lipopolysaccharide-induced lung injury, and restored pulmonary integrity. Their angiogenic potential was also attributed to activation of the RAF/ERK signaling pathway (Wu et al., 2018).

The therapeutic potential of EPC-EVs was also investigated in a diabetic wound model. Skin wound healing in control and diabetic mice was significantly enhanced by EPC-EVs administration, and immunohistochemical analyses showed that the EVs increased protein expression levels of the angiogenesis-related factors VEGF, CD31 and cell proliferation marker Ki67. In contrast to previous research, this study elucidated that the angiogenic capabilities of the EPCs-EVs were mediated through the involvement of the AGE-RAGE signaling pathway in diabetic complications (Xu et al., 2020). Jia and collaborators conducted experiments involving HUVECs and a unilateral tibial distraction osteogenesis (DO) model in Sprague-Dawley rats, where bone regeneration was notably accelerated in rats treated with EPC-EVs. Moreover, the EPC-EVs treated group exhibited higher vessel density compared to the control group, indicating effective angiogenesis stimulation during DO. *In vitro* analyses demonstrated that EPC-EVs enhanced endothelial cell proliferation, migration, downregulated SPRED1, and activated the RAF/ERK signaling pathway. Additionally, EPC-EVs upregulated the expression of angiogenesis-related genes in HUVECs, including hypoxia-inducible factor 1 (HIF-1α), VEGF, FGF-2, TGF- β1, and angiopoietin-1 (ANG1) (Jia et al., 2019).

Besides EVs originating from EPCs, it has been discovered that EVs derived from macrophages also elicit elevated levels of angiogenesis. *In vitro* experiments demonstrated increased migration, proliferation, and tube formation of HUVECs after treatment with macrophage-derived EVs. *In vivo* studies proved a reduction in diabetic wound size, accompanied by increased collagen deposition, re-epithelialization, and neovascularization (Lu et al., 2022). In another study, Lyu et al. revealed the efficacy of pro-resolving/healing macrophage derived-EVs (M2-EVs), in promoting wound healing. *In vitro* experiments demonstrated enhanced cell proliferation, wound migration, and tube formation, along with increased VEGF expression in HUVECs. *In vivo* observations showed that the wound tissues of M2-EVs treated mice had a higher level of vascularization with higher vessel density and more mature vessels than the wound tissues of control mice. Furthermore, M2-EVs induced wound healing was accompanied by increased activation of the AKT/mTOR pathway (Lyu et al., 2022).

### 4.3 Inhibition of inflammation

The application of native EVs has shown promise in inflammation inhibition, demonstrated by their ability to promote macrophage polarization towards the M2 phenotype, enhance the expression of anti-inflammatory cytokines, and reduce the expression of pro-inflammatory cytokines. During the inflammatory phase of wound healing, macrophages play a crucial role in transitioning from inflammation to proliferation. (Yunna et al., 2020). *Kim et al.* showed that M2-EVs prompt the reprogramming of macrophages from an M1 to an M2 phenotype *in vitro*, thereby completely eliminating the expression of the M1 marker ‘inducible nitric oxide synthase (iNOS)’, while simultaneously inducing the expression of the M2 marker ‘arginase-1’ Furthermore, they discovered that subcutaneous administration of M2-EVs at the wound edge successfully decreased local M1 populations and increased M2, directly converting M1 to M2 at the wound site and accelerating healing and wound closure by enhancing angiogenesis, re-epithelialization, and collagen deposition. Additionally, cytokines such as CCL24, CCL22, and milk fat globule–epidermal growth factor 8 (MFG-E8) are identified as the main EVs compounds responsible for the phenotype change (Kim et al., 2019). Beside this study, *Lyu et al.* also demonstrated that M2-EVs caused an increase in anti-inflammatory cytokine IL-10 and a decrease in anti-inflammatory cytokines IL-1β and TNF-α (Lyu et al., 2022).

Besides M2-EVs, EVs derived from keratinocytes (KC-EVs) and plasma (PL-EVs) also play a comparable role in inducing phenotypic changes in macrophages. The study of Xiaoju et al. revealed that KC-EVs display a distinct N-glycan composition on their surface, facilitating their uptake by macrophages at the wound site, causing the reprogramming of macrophages from an M1 to an M2 phenotype, downregulating pro-inflammatory genes iNOS, CD74, TNF-α, and upregulating the anti-inflammatory genes *Chemokine (C-C motif) ligand 3 (CCL3*) (Zhou X. et al., 2020). Additionally, subcutaneous administration of KC-EVs at the wound edge successfully decreased macrophage infiltration, promoted polarization from M1 to M2 at the wound site, and improved barrier function.

The study by Adamczyk and colleagues revealed that PL-EV treatment differentiated macrophages into an M2-like basal phenotype characterized by the surface expression of markers associated with the resolution of inflammation, such as CD163, CD206 and Mer Tyrosine Kinase (Adamczyk et al., 2023). Furthermore, treating macrophages with PL-EVs reduced the release of the pro-inflammatory agents IL-6 and TNF-α while enhancing the IL-10 response. This anti-inflammatory effect was linked to improved tissue repair capabilities in macrophages, as evidenced by enhanced efferocytosis and angiogenic potential of endothelial cells, and a boost in the gene expression of VEGFa, CD300e, RGS2, and CD93, all of which play roles in cell growth and tissue remodeling. Finally, the researchers discovered that PL-EVs activated the PGE2/CREB pathway, leading to a shift in macrophage phenotype to M2, reducing the production of inflammatory cytokines and enhancing tissue repair functions (Adamczyk et al., 2023).

Research on neutrophil-derived EVs (N-EVs) indicates their dual anti- and pro-inflammatory roles, influenced by environmental factors present during EV formation. Under infectious conditions, N-EVs enhance reactive oxygen species (ROS) and IL-8 production in neighboring neutrophils, while also upregulating adhesion molecules E-selectin and VCAM-1 on endothelial cells, signifying activation of these cells. Conversely, N-EVs in a resting state show no impact on endothelial function or its attenuation (Kolonics et al., 2021).

### 4.4 Enhancing skin remodeling

Skin remodeling is the final phase of the wound healing process, which follows the earlier stages of hemostasis, inflammation, and proliferation. During this phase, the initially deposited collagen in the wound site is reorganized and matured, enhancing the strength and integrity of the newly formed tissue. Key activities in skin remodeling include the breakdown of excess collagen and other ECM components that were rapidly produced during the proliferation phase. This is accomplished through enzymatic processes involving matrix metalloproteinases (MMPs). During ECM reorganization MMP-1 and MMP-8 are especially activated. *Jung Oh. et al.* investigated the effect of fibroblast-derived EVs (F-EVs) on the expression of ECM genes, and showed an increase in the mRNA expression of *MMP-1, MMP-3*, collagen I and III in other fibroblasts, which led to enhanced fibroblast migration and proliferation, and ECM reorganization. Furthermore, they showed that treatment with F-EVs has a positive effect on angiogenesis by enhancing the migration and tube formation of endothelial cells. Additionally, the combination of F-EVs with fibrin glue accelerated wound healing in a mouse skin wound model by enhancing collagen formation and maturation, and increasing blood vessel formation in the wounded skin (Oh et al., 2021). Another study showed that the action of endothelial cell derived EVs on annulus fibrosus (AF) cells causes the enhanced matrix catabolism, which consequently induced angiogenesis *in vitro*. Similarly, AF cells exposed to EVs from endothelial cells demonstrated an increase in MMP activity, marked by elevated levels of MMP-1, MMP-3, and MMP-13 at both mRNA and protein level (Pohl et al., 2016).

Additionally, the research conducted by de Jong and his team revealed that under hypoxic conditions, HMEC-1 release EVs that present lysyl oxidase-like 2 (LOXL-2), a member of the LOX family. This enzyme plays a crucial role in skin remodeling by catalyzing the crosslinking of collagen and elastin fibers, which enhances tissue strength and elasticity (de Jong et al., 2016). They showed that LOXL-2 results in fibroblast activation and subsequent ECM contraction and crosslinking.

In addition to their role in the proliferation phase, human KC-EVs also play a crucial role in tissue remodeling by initiating fibroblast differentiation (Li Q. et al., 2019). Treatment with KC-EVs promotes fibroblast migration, differentiation, and contraction, consequently inducing a pro-angiogenic response in endothelial cells. Mechanistically, KC-EVs likely target specific essential effector mRNAs in fibroblasts, such as MMP-1, MMP-3, tissue inhibitor of metalloproteinases 3 (TIMP3), and TIMP4, leading to increased MMP expression and enzymatic activities. Moreover, KC-EVs regulate α-smooth muscle actin (SMA) and N-cadherin, promoting fibroblast-to-myofibroblast differentiation. Additionally, KC-EVs promote diabetic cutaneous wound healing in rat models. Notably, KC-EVs express miR-21, which downregulates PTEN and RECK at the protein level and activates the MAPK/ERK signaling cascade, thereby enhancing fibroblast functions.

## 5 The role of mesenchymal stem cell-derived extracellular vesicles in wound healing

**FIGURE 2:**
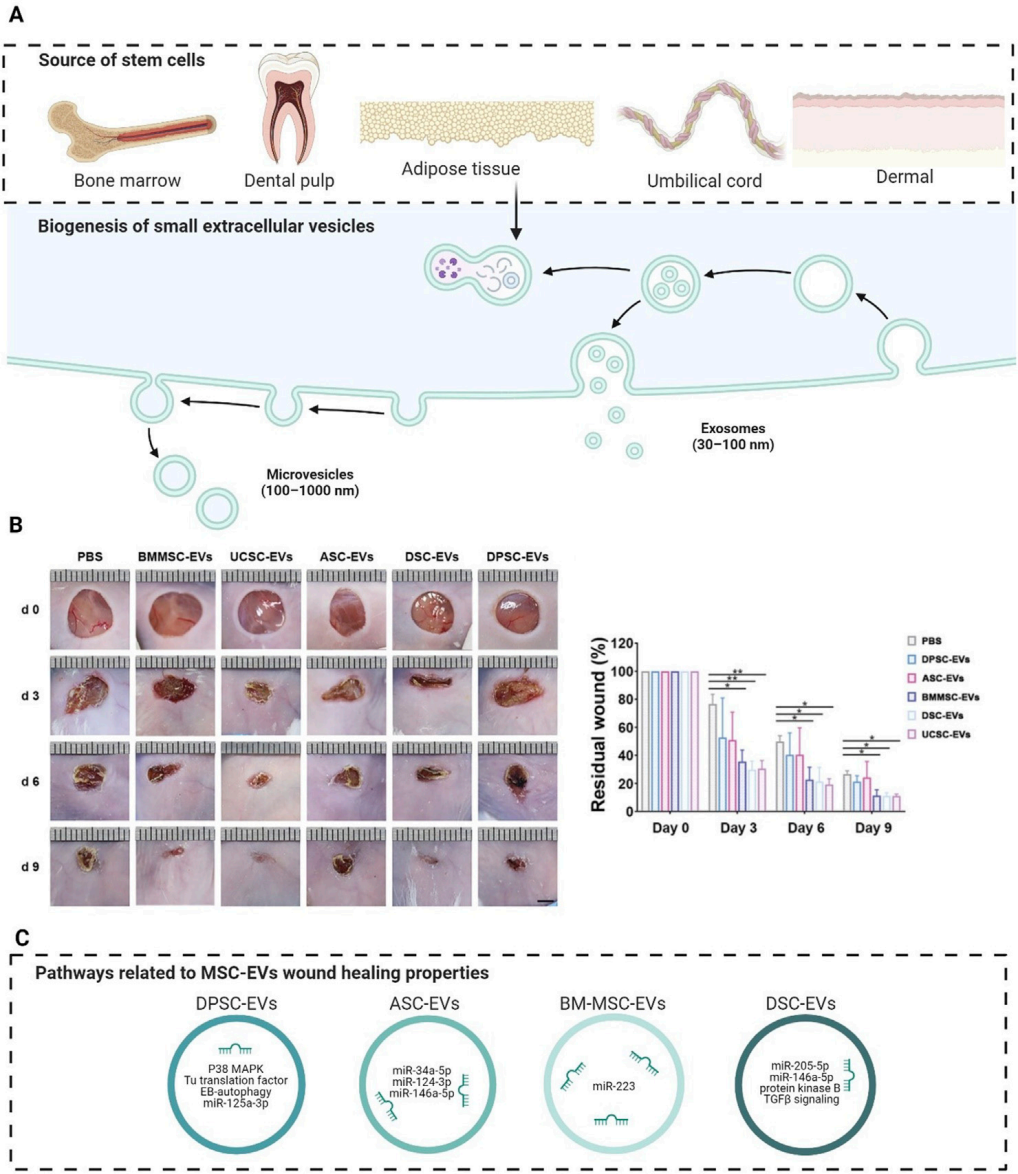
The therapeutical potential of MSC-EVs in wound healing. **(A)** MSC-EVs can be derived from several parental cells, and their regenerative properties are mainly related to small EVs (50–200 nm), which can be formed from different biogenesis routes. **(B)** Screening of the wound healing potential from different MSC-EVs sources, revealing that UCSC-EVs, BM-MSC-EVs and DSC-EVs perform better compared to EVs derived from other types of MSCs. **(C)** Each MSC-EV has its own cargo inherited from its source cell, and this can result in different pathways that lead to regeneration in wound healing. Figures A and C were created with biorender.com. Figure B was made with adaptations and permissions by (90) Abbreviations: ASCs, adipose tissue-derived stem cells; BM-MSCs, bone marrow mesenchymal stem cells; DSC, dermal stem cells; DPSCs, dental pulp stem cells; EVs: extracellular vesicles; MSCs: mesenchymal stem cells; UCSCs, umbilical cord stem cells.

As described in the introduction, EVs derived from various stem cell types and sources have garnered significant attention for their potential role in wound healing. Recently, considerable interest has arisen in the therapeutic potential of MSC-derived EVs for wound healing, highlighting their potential application in enhancing wound recovery (Kim et al., 2023). Notably, MSCs can be isolated from different tissue types, hence a broad scale of MSCs have been investigated, including dental pulp stem cells (DPSCs), apical papilla stem cells (SCAPs), adipose-tissue derived stromal cells (ASCs), bone marrow-derived stem cells (BM-MSCs), umbilical cord stem/stromal cells (UCSCs), and dermal-derived stem cells (DSCs), collectively representing a versatile toolkit for advancing wound care and regenerative medicine (Figure 2A). Their bioactive cargo influenced by their biogenesis and parental cells can modulate cellular processes such as proliferation, migration, and differentiation, which are crucial for tissue regeneration and wound repair. They have shown promise in promoting healing by modulating inflammation, enhancing cell communication, and stimulating tissue regeneration. The therapeutic potential of various sources of MSC-EVs was recently evaluated using full-sickness skin defect mouse models and local administration. BM-EVs, UCSC-EVs, and DSC-EVs demonstrated superior wound healing outperforming ASC-EVs and DPSC-EVs, evidenced by reduced wound areas and faster closure rates from day 3 to day 9, as seen in Figure 2B (Li et al., 2023). Although their mode of action was not explored, their screening shows the importance of a rational strategy to select the most suitable MSC-EVs source for clinical translation. In the following sections, we describe in detail the role and mode of action of different MSC-EVs in different phases of wound healing (Overview is shown in Figure 2 and Table 2).

**TABLE 2:** The role of mesenchymal stem cell extracellular vesicles in wound healing.

Phase	EVs source	Model/in vitro	Effect/in vivo	Signaling pathway/Factor	Reference
**Hemostasis**	UCSCs	**Human blood and platelet-free plasma:** ↓Blood clot formation, ↓lag period of spontaneous clotting, ↑clot firmness, ↑blood clot area, ↑annexin V, ↑coagulation proteins: CD9, PS, myosin-9, talin-1, histones, and cytoplasmic actin	**NE**	**NE**	Silachev et al. (2019)
	ASCs and BM-MSCs	**ASCs and BM-MSCs:** Thrombogenic and expressed higher amounts of phosphatidylserine, ↑ clotting rates, ↑the total amount of generated thrombin	**NE**	The expression of TF and PS on the surface of the vesicles	Chance et al. (2019)
	ASCs and BM-MSCs	**Whole human blood or human platelet-poor plasma:** ↑Procoagulant activity	**NE**	**NE**	Christy et al. (2017)
	ASCs	**Reference plasma (citrated human plasma), factor XII-deficient plasma, and factor VII-deficient plasma:** Factor VII-deficient plasma: ↓clot formation Factor XII-deficient plasma: prolonged clotting times	**NE**	Coagulation pathway	Fiedler et al. (2018)
**Proliferation and angiogenesis**	SCAPs	**HUVECs:** ↑Migration, ↑proliferation, ↑Angiogenesis, ↑angiogenesis-related gene expression of CD31	**C57BL/6J mice gingival excisional wound model** wound area of the palatal gingiva was significantly smaller,↑Blood vessel formation	Cdc42	Liu et al. (2021)
	Periodontally compromised teeth DPSCs and Patient-matched DPSCs from periodontally healthy teeth	**HUVECs:** ↑Migration, ↑proliferation, ↑tube formation, ↑angiogenesis, ↑angiogenesis-related gene expression of VEGF and AngII	**C57BL/6 mice excisional wound model** Accelerated cutaneous wound healing in mice, ↑vessel formation in the wound sites of mice	Not determined	Zhou et al. (2020b)
	DPSCs isolated from third molars	**HUVECs:** ↑Migration, ↑proliferation, ↑tube formation, ↑angiogenesis, ↑angiogenesis-related protein expression of VEGF, induced apoptosis during the initial stage of angiogenesis	**NE**	Not determined	Zhang et al. (2020)
	DPSCs isolated from third molars and premolars	**HUVECs:** ↑Angiogenesis-related gene/protein expression of VEGF, AngII, CD31, FGF2, CD144, KDR, SDF-1, CXCR4, and MMP-9, ↑migration, ↑proliferation, ↑tube formation, ↑angiogenesis	**NE**	p38 MAPK signaling inhibition	Xian et al. (2018)
	ASCs	**HUVECs:** ↑Proliferation, ↑migration, ↑tube formation	**C57BL/6J mice excisional wound model** Accelerated wound healing ↑CD31, CD206 expression and pAkt phosphorylation	miR-125a-3p and PTEN	Pi et al. (2022)
	UCSCs	**HaCaT:** ↑Migration, ↑proliferation, ↑suppression of apoptosis, ↑PARP-1, ↑PAR	**BALB/d mice Full-thickness excisional wound model** ↑Epidermal re-epithelialization, ↑dermal angiogenesis, ↑wound closure, ↑CK10, CD31 expression, ↓α-SMA	PARP-1 and AIF apoptosis pathway	Zhao et al. (2020)
	UCSCs	**EA.hy926:** ↑Migration, ↑proliferation, ↑tube formation, ↑Cyclin D3, ↑N-cadherin, ↑β-catenin, *↓E-cadherin*	**Albino wistar rats skin burn model** Accelerated wound healing, ↑ angiogenesis,	Wnt4/β-catenin pathway	Zhao et al. (2020)
	BM-MSCs	**EA.hy926, HaCat, TERT-1 keratinocytes:** ↑Migration, ↑proliferation, ↑angiogenesis, ↑MMP13 *↓*type I,and III collagen, *↓*α-smooth muscle actin, *↓*MMP2, *↓*MMP14,	**NE**	Not determined	Tutuianu et al. (2021)
	BM-MSC and DPSCs	**HUVECs:** ↑Migration, ↑angiogenesis, ↑proliferation **EVs derived from BM-MSCs:** ↑In ovo angiogenesis, ↑IGFBP-1, IGFBP-3, and TIMP1 **EVs derived from DPSCs:** Angpt-1	**NE**		Merckx et al. (2020)
**Inflammation**	ASCs	**Dermal fibroblasts treated with proinflammatory cytokines IFN-γ and TNF-α:** ↑Arg1 and ↑CD206 M2 markers, ↓TNF-α, ↓IL-6, ↓IL-8 and ↑IL-10 **Fibroblast:** ↑Fibroblast proliferation, ↑fast closure of the scratch	**NE**	miR-34a-5p, miR-124-3p miR-146a-5p	Heo et al. (2021)
	BM-MSCs and jaw BM-MSCs	**NE**	**C57BL/6J mice excision wound model** ↑CD206 M2 marker, ↑IL-10 and ↓TNF-α,↑wound closure	miR-223	He et al. (2019)
	BM-MSCs	**Macrophages RAW264.7 cells:** ↑Arg1 and ↑IL-10 M2 markers, ↓IL-1β, ↓TNF-α, ↓iNOS	**Sprague-Dawley rats diabetic- excisional wound model** ↑Angiogenesis, ↑collagen synthesis	Not determined	Liu et al. (2020)
	DPSCs isolated from third molars	**Macrophages RAW 264.7 cells:** ↑CD206 M2 markers, ↓TNF-α, ↓IL-6, ↓IL-1β, and ↑IL-10, ↑IL-1ra	**NE**	miR-125a-3p	Zheng et al. (2020)
**Skin remodeling**	DPSCs isolated from third molars	**EV-Hydrogel of** **fibrinogen from human plasma:** Promoted deposition of collagen I, III, and IV	**NE**	Not determined	Zhang et al. (2020)
	ASCs	**Skin fibroblasts:** ↑Cell migration, proliferation, ↑collagen synthesis, ↑expression of N-cadherin, cyclin-1, PCNA and collagen I, III.	**BALB/c mice excisional wound model** Accelerated cutaneous wound healing, ↑collagen I and III production, ↓scar formation	Optimizing the characteristics of fibroblasts	Hu et al. (2016)
	ASCs	**NE**	**BALB/c mice excisional wound model** ↓The size of scars, ↑ratio of collagen III to collagen I in murine incisional wounds, ↑the ratio of transforming growth factor-β3 (TGF-β3) to TGF-β1, ↑matrix metalloproteinases-3	ERK/MAPK pathway	Wang et al. (2017)
	UCSCs	**Skin fibroblasts:** ↓TGF-β-Induced myofibroblast formation	**Nude mice (BALB/c-ν) excisional wound model** ↓Myofibroblast aggregation and scar formation	miR-21, -23a, −125b, and −145. Inhibition of the β2/SMAD2 pathway	Fang et al. (2016)
	UCSCs	**Human keratinocytes:** ↑Proliferation, ↑collagen synthesis	**Sprague–Dawley rats second-degree burn wound model** Restricted excessive skin cell expansion and collagen deposition during the tissue remodeling period of cutaneous regeneration. Amplified epidermal and dermal cells and enhanced re-epithelialization	Wnt/β-catenin signaling	Zhang et al. (2016b)
	iPSCs	**HUVECs:** ↑Proliferation, ↑migration, ↑tube formation, ↑angiogenesis, promoted deposition of collagen type I and II	**Sprague–Dawley rats excisional wound model** ↑Wound closure areas, accelerated re-epithelialization, reduced scar widths, and the promotion of collagen maturity. Accelerated the maturation of new formed vessels in wound sites	NE	Zhang et al. (2015b)

AGE-RAGE, Advanced Glycation End-products and Receptor for Advanced Glycation End-products; AngII, Angiotensin II; ANG1, Angiopoietin 1; ANG2, Angiopoietin 2; Arg1, Arginase 1; ASCs, Adipose-derived Stem Cells; BM-MSCs, Bone Marrow-derived Mesenchymal Stem Cells; CD206, Cluster of Differentiation 206; Cdc42, Cell Division Cycle 42; CXCR4, C-X-C Chemokine Receptor Type 4; DPSCs, Dental Pulp Stem Cells; EA.hy926, Human Umbilical Vein Endothelial Cell-Derived Cell Line; EB, Eukaryotic Initiation Factor 2-associated Transcription Factor; ERK, Extracellular Signal-Regulated Kinase; EVs, Extracellular Vesicles; FGF-2, Fibroblast Growth Factor 2; HaCaT, Human Adult Low Calcium High Temperature Keratinocytes; HIF-1α, Hypoxia-Inducible Factor 1-alpha; hucMSCs, Human Umbilical Cord Warton’s Jelly Mesenchymal Stem Cells; IGFBP-1, Insulin-like Growth Factor Binding Protein 1; IGFBP-3, Insulin-like Growth Factor Binding Protein 3; IL-1β, Interleukin 1 beta; IL-1ra, Interleukin 1 Receptor Antagonist; IL-6, Interleukin 6; IL-8, Interleukin 8; IL-10, Interleukin 10; iNOS, Inducible Nitric Oxide Synthase; iPSC, induced pluripotent stem cell-derived mesenchymal stem cells; KDR, Kinase Insert Domain Receptor (VEGFR-2); MAPK, Mitogen-Activated Protein Kinase; MMP-2, Matrix Metalloproteinase 2; MMP-9, Matrix Metalloproteinase 9; N-cadherin, Neural Cadherin; NE, Not Evaluated; NF-κB, Nuclear Factor kappa-light-chain-enhancer of activated B cells; PAR, Poly (ADP-ribose); PARP-1, poly (ADP ribose) Polymerase; PCNA, Proliferating Cell Nuclear Antigen; SCAPs, Apical Papilla Stem Cells; SD, Sprague-Dawley; SDF-1, Stromal Cell-derived Factor 1; TGF-β1, Transforming Growth Factor Beta 1; TGF-β3, Transforming Growth Factor Beta 3; TIMP-1, Tissue Inhibitor of Metalloproteinases 1; TNF-α, Tumor Necrosis Factor-alpha; Tu, Translation Elongation Factor Tu; UCSCs, Umbilical Cord Stem Cells; VEGF, Vascular Endothelial Growth Factor; Wnt/β-catenin, Wingless-related integration site/β-catenin signaling pathway; ↑, increased; ↓, decreased.

### 5.1 Promoting hemostasis

As described above, wound healing begins with blood clot formation, which prevents infection and protects against blood loss. This dynamic process relies on platelet aggregation. MSC-derived EVs are known to have procoagulant properties. Silachev et al. demonstrated that UCSC-EVs significantly reduce human blood clot formation time and the lag period of spontaneous clotting compared to the untreated group (Silachev et al., 2019). Additionally, the UCSC-EV group showed improved clot firmness and a significantly increased blood clot area. Proteomic analysis revealed that both cells and UCSC-EVs contain several well-known coagulation proteins, including CD9, PS, myosin-9, talin-1, histones, and cytoplasmic actin. Furthermore, they found that UCSC-EVs contain annexin V, a protein known for its anticoagulant activities. Additionally, Chance et al. investigated whether EVs isolated from three-dimensional cultures exhibit anticoagulant activities, alongside the presence of factors associated with procoagulant activity (Chance et al., 2019). The researchers assessed the procoagulant activity of EVs derived from ASCs and BM-MSCs. Both EV groups displayed functional thrombogenicity, significantly increasing peak thrombin activity. Moreover, all EVs showed a substantial increase in the total amount of generated thrombin. The study also confirmed that the procoagulant activity of EVs is linked to the expression of tissue factor (TF) and PS on the surface of the vesicles. Similarly, another study comparing ASC-EVs and BM-EVs confirmed that ASC-EVs exhibit higher procoagulant activity in whole human blood or human platelet-poor plasma, correlating with their expression levels of TF (Christy et al., 2017).

Furthermore, Fiedler et al. investigated EVs from unstimulated ASCs and those treated with lipopolysaccharide (LPS) and TNF-α, conducting clotting experiments with these EVs compared to reference plasma (citrated human plasma), factor XII-deficient plasma, and factor VII-deficient plasma (Fiedler et al., 2018). In factor VII-deficient plasma, no clot formation was observed with any EV group, suggesting the presence of TF in EVs, which activates the extrinsic pathway of coagulation dependent on factor VII. In factor XII-deficient plasma, both unstimulated and TNF-α-treated EVs showed significantly prolonged clotting times, possibly indicating the presence of PS molecules on EVs, which facilitate factor XII activation and stimulate the intrinsic coagulation pathway. This suggests that ASC-EVs may contribute to wound healing through various pathways independent of pro-inflammatory stimuli.

### 5.2 Enhancing proliferation and angiogenesis

Adequate formation of new blood vessels, also known as angiogenesis is a key step in wound healing and numerous studies have shown that this process is enhanced by EVs.

For example, EVs derived from DPSCs were found to promote key steps in the angiogenic cascade such as endothelial cell proliferation, migration and tube formation. A recent study compared the angiogenic potential of human periodontally compromised teeth (P-DPSCs) and patient-matched DPSCs derived from periodontally healthy teeth (H-DPSCs) (Zhou H. et al., 2020). Conditioned medium (CM) of both DPSC types induced this process and the proangiogenic effects were inhibited in CM derived from DPSCs where EV secretion was blocked by pretreatment with GW4869. In addition, P-DPSC-EVs led to higher expression levels of angiogenesis-related genes and proteins (VEGF and angiotensin II) in endothelial cells compared to H-DPSC-EVs. Similarly, both P-DPSC-EVs and H-DPSC-EVs were found to accelerate wound healing and promote vascularization across skin defects in mice, but wounds treated with P-DPSC-EVs showed quicker healing and enhanced new vessel formation.

Siyuan et al. discovered similar outcomes when they monitored the effects of DPSC-EVs on endothelial cell proliferation and migration in fibrin hydrogels (Zhang et al., 2020). DPSC-EV-fibrin gels promoted HUVEC proliferation, migration, and tube formation and facilitated vascular-like structure formation by increasing the VEGF release. Additionally, the DPSC-EV-fibrin gel facilitated the deposition of collagen I, III, and IV, and readily induced apoptosis during the initial stage of angiogenesis. Another study concluded that DPSC-EVs facilitated endothelial cell proliferation and increased tube formation and proangiogenic factor gene/protein expression of VEGF, AngII, CD31, FGF-2, CD144, KDR, SDF-1, CXCR4, and MMP-9. Furthermore, they demonstrated that inhibition of p38 mitogen-activated protein kinase activation enhanced DPSC-EVs stimulated tubular morphogenesis (Xian et al., 2018).

Other researchers have explored the impact of SCAP on critical-size defects in maxillofacial soft tissue (Liu et al., 2021). SCAP-derived EVs promoted vascularization to accelerate tissue regeneration of the palatal gingiva in mice. The wound area of the palatal gingiva was significantly smaller in the SCAP-derived EVs infusion group at 3 and 7 days post-wounding than in the control group. Furthermore, the expression of the angiogenic protein CD31 was increased in a dose-dependent manner. SCAP-derived EVs facilitated the transfer of Cdc42, a key factor in restructuring the cell framework, which enhanced both the proliferation and migration of endothelial cells. This transfer was pivotal in prompting vascularization during the wound healing process. Additionally, in the subcutaneously implanted matrigel model, the SCAP-derived EV group had more blood vessels than the control group. A recent study compared the angiogenic actions of DPSC-EVs with BM-MSC-EVs by using a protein array (Merckx et al., 2020). DPSC-EVs displayed higher concentrations of ANG1, while there were no differences in VEGF levels. Both EV types attracted HUVECs in the Boyden chamber transwell assay.

BM-MSC-EVs were found to stimulate endothelial cell tube formation and migration *in vitro* and to induce wound healing in a skin organotypic model, which exhibited full re-epithelialization upon EV exposure (Tutuianu et al., 2021). Two different studies reported that UCSC-EVs enhanced angiogenesis (Zhao et al., 2020; Zhang B. et al., 2015). Zhang *et al.* reported that UCSC-EVs induced proliferation, migration and tube formation of endothelial cells in a dose-dependent manner and enhanced wound healing and angiogenesis in a rat skin burn model; This effect was mediated by the Wnt4/β-catenin pathway as knockdown of Wnt4 inhibited the *in vivo* proangiogenic effects (Zhang B. et al., 2015). ADSC-EVs also promote angiogenesis *in vitro* and wound healing in mice and this effect was inhibited by knockdown of miR-125a-3p (Pi et al., 2022).

### 5.3 Inflammation

During wound healing, inflammation is a natural and crucial phase, playing a pivotal role in the defense and tissue repair of the skin. However, an excessive or prolonged inflammatory response can impede wound healing (Wang Z. et al., 2022; Kotwal and Chien, 2017). This process is where MSC-derived EVs step in, regulating inflammation to optimize wound healing. The brilliance of MSC-derived EVs lies in their ability to promote the transition of M1 macrophages to the M2 phenotype, orchestrating an anti-inflammatory environment conducive to tissue repair and optimal wound healing outcomes (Heo et al., 2021; He et al., 2019; Liu et al., 2020; Tsutsui, 2020; Tatullo et al., 2015). June *et al.* used dermal fibroblasts treated with pro-inflammatory cytokines IFN-γ and TNF-α as an inflammatory model to examine the effect of ASC-EVs (Heo et al., 2021). They showed that ASC-EVs-treated fibroblasts showed the most significant increase in expression of arginase 1 (Arg-1) and CD206, markers of M2 polarized macrophages, compared with untreated fibroblasts. Similarly, the research group of Bei Li *et al.* discovered that BM-MSC-EVs stimulated macrophage polarization from M1 to M2 (He et al., 2019). In this scenario, BM-MSCs could migrate to the wound site, induce M2 macrophage polarization, and consequently enhance wound healing. *In vitro* coculture experiments of MSCs with macrophages showed elevated levels of the M2 macrophage marker CD206, illustrating the ability of BM-MSC-EVs to drive macrophage transformation. Conversely, the depletion of BM-MSC-EVs decreased the expression of the M2 markers in macrophages. Liu et al. recently characterized the macrophage phenotype change by melatonin-treated BM-MSC-EVs in a RAW264.7 cell line (Liu et al., 2020). They showed that the M2 macrophage markers of anti-inflammatory IL-10 and Arg1 gene expression were raised after EV treatment. Another remarkable feature of MSC-EVs is the reduction of the production of inflammatory cytokines. For example, when inflammatory cytokine-stimulated fibroblasts were treated with ASC-EVs, there was a reduction in the levels of the pro-inflammatory proteins TNF-α, IL-6, and IL-8, alongside an increase in the anti-inflammatory IL-10 expression. Additionally, Xiaoning et al. discovered a reduction in the levels of the pro-inflammatory proteins TNF-α, alongside an increase in the anti-inflammatory IL-10 expression in the BM-MSC-EVs treated group. Consequently, gene expression of proinflammatory IL-1β and TNF-α was significantly decreased.

In addition, DPSC-derived factors have been shown to induce the conversion of M1-type macrophages to the M2 phenotype. Jianmao et al. investigated the effects of DPSC-EVs on macrophage polarization (Zheng et al., 2020). They showed that DPSC-EVs switched macrophages from M1 to the pro-healing M2 phenotype by inhibiting toll-like receptor (TLR) and NFκΒ signaling. miRNA sequencing found 81 miRNAs significantly altered in DPSC-EVs, with miR-125a-3p showing a 12-fold upregulation. Exosomal miR-125a-3p switched macrophages toward the M2 phenotype via inhibiting NFκΒ and TLR signaling via direct IKBKB targeting. Additionally, this study discovered a reduction in the levels of the pro-inflammatory proteins TNF-α, IL-6 and IL-1β, alongside an increase in the anti-inflammatory IL-10 and IL-1ra expression in the DPSC-EV treated group.

### 5.4 Enhancing skin remodeling

Early-stage tissue restoration is pivotal in successful healing, emphasizing rapid wound closure and collagen production and maturation. Siyuan et al. demonstrated the fabrication of a DPSC-EVs-fibrin gel composite as an *in situ* forming delivery system (Zhang et al., 2020). They found that the EV-fibrin gel facilitated the deposition of collagen types I, III, and IV. By promoting the deposition of these collagen types, DPSC-EVs contribute significantly to the formation and maturation of the ECM, which is crucial for tissue restructuring and strength. Another study also found the same effect in ASCs, which promoted skin regeneration by increasing the synthesis of collagen I and III in the early stages of wound healing, while reducing scar formation by inhibiting collagen expression (Hu et al., 2016). Furthermore, Wang et al. found that ASC-EVs can serve as a therapeutic tool for scarless wound healing by preventing fibroblast differentiation into myofibroblasts and altering the ratios of several cellular molecules, including collagen type III to collagen type I, MMP-3 to MMP-1, and TGF-β3 to TGF-β1 (Wang et al., 2017). Also, induced pluripotent stem cell-derived MSC EVs (iPSC-EVs) have been found to increase the mRNA expression of type I and III collagen, and elastin, which enhances collagen maturation and reduces scar width by increasing iPSC-EVs concentration (Zhang J. et al., 2015). Furthermore, UCSC-EVs was shown to positively affect skin remodeling by inhibiting the TGF-β/SMAD2 signaling pathway (Fang et al., 2016). This pathway is important for this stage in angiogenesis, because it regulates the balance between fibroblast differentiation and myofibroblast formation. By preventing excessive differentiation into myofibroblasts, it maintains proper ECM composition and promotes the formation of new blood vessels, which are crucial for effective wound healing and tissue regeneration. Additionally, UCSC-EVs cargo contain the 14-3-3ζ protein, which enhances YAP phosphorylation by regulating the binding of YAP substrates to p-LATS kinase, thereby improving repair of the damaged tissue (Zhang B. et al., 2016).

## 6 Epidermal stem cell-derived extracellular vesicles in wound healing

Epidermal stem cells (ESCs) are a specific type of stem cell found in the epidermis, the outermost layer of the skin. These cells reside in the basal layer of the epidermis and play a fundamental role in maintaining the skin’s homeostasis and regenerative capacity. Epidermal stem cells have the remarkable ability to self-renew and generate various types of cells that make up the skin, including keratinocytes. The EVs of stem cells are critical for skin regeneration, wound healing, and maintaining the continuous turnover of skin cells, ensuring that the skin remains functional and can effectively regenerate after injuries or damage.

Peng et al. explored the functions of EVs secreted by ESCs (ESC-EVs) *in vitro* and ultimately assessed their remarkable reparative capacity in a type 2 diabetic mouse model of wound healing (Wang P. et al., 2022). ESC-EVs enhanced the proliferation and migration of fibroblasts and macrophages and promoted alternative or M2 macrophage polarization *in vitro*. In wounds of db/db mice, treatment with ESC-EVs accelerated wound healing and wound closure by decreasing inflammation, augmenting wound cell proliferation, stimulating angiogenesis, and inducing M2 macrophage polarization. In addition, ESC-EVs led to higher expression levels of angiogenesis-related genes/proteins such as endoglin, CD31, PLGF-2, VEGF-A, and TGFβ3. ESC-EVs regulate TGFβ signaling, which is the pathway most responsible for wound healing. *In silico* functional analysis showed that the ESC-EVs microRNAs‒target genes were primarily involved in homeostatic processes and cell differentiation and highlighted regulatory control of phosphatidylinositol-3 kinase/protein kinase B and TGFβ signaling pathways, which was further validated by *in vitro* studies.

## 7 Clinical translation of EVs in wound repair

With the proven therapeutic potential of EVs in wound repair, several key steps are needed to translate their potential to clinical setups. The main challenge lies in large-scale manufacturing, as therapeutic applications require approximately 10^13^ EVs per dose, while traditional culture methods typically yield only 10^9^–10^11^ EVs per liter of conditioned medium (Cully, 2021). To address this, allogeneic EVs are preferred over autologous EVs due to their greater scalability and standardization potential and direct availability to the patient (as autologous cell culture and EV production are time-consuming). A major drawback of allogeneic EVs is the potential for immunogenic response compared to autologous EVs (Dong et al., 2020). In this context, a study in rhesus macaques comparing directly allogenous and autologous iPSC-EVs showed that both types induced wound healing without triggering immune response, while autologous EVs being more effective their allogenic counterparts. It is known that the EVs of certain cell types such as tumor cells, macrophages but also red blood cells trigger immune responses because of their cargo (e.g., they can contain pro-inflammatory cytokines and several DAMPs such as heat-shock proteins) (Buzas, 2023). Interestingly, a recent systematic review of clinical trials on EVs reported a higher incidence of adverse effects with autologous EVs compared to allogeneic ones. However, the clinical relevance of this finding remains unclear, highlighting the need for further investigation to determine its impact on therapeutic outcomes (Van Delen et al., 2024). As discussed in this review, EVs from MSCs have potent anti-inflammatory and immunomodulatory capacities, making them an ideal source for an ‘off-the-shelf’ allogenic wound treatment (Lu et al., 2019).

In the optimization of manufacturing processes for EVs, their heterogeneity is influenced by factors such as cell source, bioreactor type, culture conditions, and isolation methods, as detailed in these references (Pincela Lins et al., 2023; Li et al., 2025). Recently, a clinical trial in EV therapy for wound healing focused on developing clinical-grade P-EVs. In this study, the ligand-based exosome affinity purification (LEAP) method enabled the production of P-EVs with consistent size and cargo, thereby improving scalability and purity. *In vitro* assays demonstrated that LEAP-purified P-EVs enhanced fibroblast proliferation, migration, and angiogenesis. Although LEAP-produced P-EVs showed no significant difference in mean healing time compared to placebo-treated wounds, they demonstrate promise as a scalable method for producing consistent, clinical-grade EVs for effective wound healing (Johnson et al., 2023).

## 8 Conclusions and future perspectives

EVs of native skin and immune cells are important messengers during all steps of wound healing, from hemostasis to skin remodelling. In each step, one type of cell has a main role; e.g., in hemostasis, EVs released from thrombin-activated platelets increase the formation of fibrin. There is extensive evidence that EVs derived from MSC, as well as from ESCs, have beneficial actions in all steps of the wound healing process, displaying high biocompatibility and strong bioactivity, thereby providing promising therapeutic avenues for chronic wounds. Stem cell EVs promote hemostasis, enhance cell proliferation and angiogenesis, increase the synthesis of the ECM, reduce scar formation, and are endowed with anti-inflammatory capacities. Compared with the use of cells themselves, the advantages of EVs are that they are less likely to provoke an immune response, do not cause malignant transformation, do not need a long follow-up and are easier to store and transport.

Despite accumulating evidence of the benefits of EVs, clinical translation is hampered by challenges in the heterogeneity of EVs and large-scale manufacturing. As EV preparations are heterogeneous in terms of origin, and source cells (especially for MSCs), poor reproducibility is shown due to the lack of standardization in protocols. MISEV guidelines were created, aimed at improving standardization (Thery et al., 2018). Nevertheless, the field is faced with difficulties in reproducibility and comparison between different studies by a general lack of transparency in reporting and adherence to these guidelines. Another hurdle is achieving substantial amounts of Evs. It is estimated that a clinical dose require at least 100 times more EVs than what current upscaling and isolation methods can produce (Cully, 2021). Innovative solutions to upscale, such as bioreactors and advanced purification techniques (as asymmetrical flow filtration), can boost production but are costly and not yet widely accessible. Upscaling methods are shown to alter EV bioactivity, so further research is needed (Guo et al., 2021).

To further improve the function and targeting of EV therapies, the study of their mode of action is paramount. Fundamental advances have been made to understand the active cargo of EVs, including proteins, miRNA, and mRNA species, which modify inflammatory and repair responses of the target cells. While the role of miRNA is thoroughly investigated, the role of proteins, lipids, and also the protein corona in EVs warrants further investigation (Wolf et al., 2022). A new promising direction in wound healing EV research is the combination of EVs with biomaterials (e.g., hydrogels), which serve as carriers enhancing EV half-life, prolonging EV bioactivation, and facilitating slow EV release, which can improve their half-life and their bioactivity (Ye et al., 2023).

In conclusion, there is overwhelming evidence that EVs derived from native cells from the wound area as well as of various stem cell types have beneficial effects on the wound healing process, showcasing the tremendous therapeutic potential of these remarkable vesicles. Further research should focus on the scalable production of EVs, and the combination of EVs with biomaterials to ensure translation to the clinic.
